# Lymphatic remapping by long-term lymphoscintigraphy follow-up in secondary lymphedema after breast cancer surgery

**DOI:** 10.1038/s41598-023-50558-7

**Published:** 2024-01-06

**Authors:** Garam Hong, Koeun Lee, Sangwon Han, Jae Yong Jeon

**Affiliations:** 1 Department of Rehabilitation Medicine, Asan Medical Center, University of Ulsan College of Medicine, 88, Olympic-Ro 43-Gil, Songpa-gu, Seoul, 05505 Republic of Korea; 2 Department of Nuclear Medicine, Kangbuk Samsung Hospital, Sungkyunkwan University School of Medicine, Seoul, Republic of Korea; 3 Department of Nuclear Medicine, Asan Medical Center, University of Ulsan College of Medicine, 88, Olympic-Ro 43-Gil, Songpa-gu, Seoul, 05505 Republic of Korea

**Keywords:** Cancer, Breast cancer

## Abstract

The purpose of the study is to investigate long-term changes on lymphoscintigraphy and their association with clinical factors in breast cancer-related lymphedema (BCRL) patients. This single-center cohort study included BCRL patients who underwent baseline and follow-up lymphoscintigraphy. The percentage of excessive circumference (PEC) of the affected upper limb compared with the unaffected side was used as an indicator of the clinical severity of BCRL. Each ^99m^Tc-phytate lymphoscintigraphy image was categorized according to the Taiwan lymphoscintigraphy staging system. Clinical parameters and the lymphoscintigraphy stage at baseline and follow-up were compared and analyzed. Eighty-seven patients were included. Baseline and follow-up lymphoscintigraphies were performed at median 7 (interquartile range [IQR]: 2‒14) and 78 (IQR: 49‒116) months after surgery, respectively. Both lymphoscintigraphy stage and PEC showed variable change with overall increases in their severity. Stepwise multivariable analysis revealed follow-up lymphoscintigraphy stage (*P* = 0.001) to be independent variables for PEC at follow-up, however, baseline lymphoscintigraphy stage was not. The clinical courses of BCRL and patients’ lymphoscintigraphy patterns showed diverse changes over long-term follow-up. In addition to initial lymphoscintigraphy for diagnosis, lymphatic remapping by follow-up lymphoscintigraphy can be useful to visualize functional changes in the lymphatic system that may guide the optimal management in BCRL.

## Introduction

Breast cancer-related lymphedema (BCRL) is a debilitating disease and often requires lifelong compression therapy^1,2^. It can occur after sentinel lymph node biopsy or axillary lymph node dissection, with reported incidences of 6% and 20%, respectively^3^. When axillary lymph nodes are removed or damaged during surgery and radiation therapy, failure of the lymphatic drainage system can result in excessive accumulation of lymphatic materials in the interstitial space, which could cause a number of morbidities such as pain, chest tightness, and impaired range of motion in the affected arm, as well as long-term complications such as cellulitis or lymphangiosarcoma^4–9^. BCRL negatively affects the patient’s overall quality of life in both physical and psychological aspects.

Clinical diagnosis of BCRL is based on physical examination, and various imaging studies which are primarily focused on assessing the volume, components, or lymphatic flow of the affected limb^10^. Circumference and volume measurement of the affected limb, with comparison between the affected and non-affected limbs, are traditional volume-based approaches for indicating the presence of BCRL^11^. Component analysis includes bioelectrical impedance analysis (BIA), computed tomography (CT), dual-energy X-ray absorptiometry, and magnetic resonance imaging (MRI)^12^. Lymphatic flow-based assessment includes lymphoscintigraphy and indocyanine green lymphography^13^. Although indocyanine green lymphography has gained clinical attention, it is difficult to visualize the deep lymphatic system with this approach because of its limited penetration depth^13^. Lymphoscintigraphy is a first-line diagnostic imaging tool for BCRL and allows visualization of the lymphatic flow and functional lymph node^14,15^. Staging systems for lymphoscintigraphy that reflect the degree of lymphatic obstruction have been proposed, and the resulting stages are reported to correlate with the clinical severity of arm lymphedema^16,17^.

The clinical courses of BCRL vary, and show functional changes in the lymphatic system including loss of pre-existing lymph node function, creation of a new collateral lymphatic pathway or peripheral lymphovenous anastomosis, and development of new dermal backflow^18,19^. As a tailored rehabilitation program may be required according to the different lymphatic drainage patterns understanding the functional changes in lymphatic circulation is important for optimal treatment^20,21^. Lymphoscintigraphy can be used to evaluate such changes, but this modality is primarily limited to diagnostic purposes at initial work-up, and little has been reported on longitudinal changes in lymphatic circulation visualized on lymphoscintigraphy^22^. Furthermore, whether changes in lymphoscintigraphic stage are related to the clinical presentation of BCRL remains unanswered. Therefore, this study aimed to investigate the relationships between long-term changes in patterns of lymphoscintigraphy and clinical features in patients with BCRL.

## Materials and methods

### Study design, setting, and patients

This is a single-center retrospective study of patients with BCRL who underwent lymphoscintigraphy at our institution between March 2008 and May 2022. Patients were identified from a review of their electronic medical records performed by the authors. All identified patients were evaluated for study eligibility. The inclusion criteria were as follows: (1) secondary lymphedema after breast cancer surgery, and (2) patients who underwent baseline and follow-up lymphoscintigraphy. The diagnosis of BCRL was made based on the clinical history, physical examination and imaging work-up. Follow-up lymphoscintigraphy was performed when persistent lymphedema was observed after the first lymphoscintigraphy. If follow-up lymphoscintigraphy was performed more than once, we chose the lymphoscintigraphy with a longer follow-up period or that with no intervening axillary surgery between baseline and follow-up lymphoscintigraphy. Patients were excluded from the analysis if (1) intervening axillary node dissection, (2) surgical lymphovenous anastomosis and/or lymph node transplantation were performed, or (3) bilateral lymphedema being present. The number of patients included during the study period determined the sample size of the study.

Demographic data including age, sex, body mass index (BMI), T and N stages of breast cancer, type of surgery and axillary node dissection, radiation therapy, and chemotherapy were retrospectively obtained via medical chart review. All patients included in the study received conservative treatment for BCRL in the interval. The types of radiation therapy were divided into nodal and non-nodal radiation. Other potential clinical risk factors including compliance, the type of compression (i.e., stockings, bandages), implementation of CDT (complex decongestive therapy), and occurrence of cellulitis were recorded. A poor compliance group was defined as those patients noted as having poor compliance in the electronic hospital record or not performing compression despite a compression-dependent status.

### Evaluation of arm circumference

The circumference of the upper limb was measured at multiple locations selected from 5, 10 cm above or 5, 10, 15 cm below, the lateral epicondyle level of the elbow at each outpatient visit during follow-up, and these measurements were noted in the electronic medical record. Circumference measurements were conducted once at each aforementioned anatomical location by a skilled physical medicine and rehabilitation specialist (JJY). The medical record of the outpatient visit that was closest to the time point of each lymphoscintigraphy was reviewed for this study. The percentage of excess circumference (PEC) was calculated as follows: PEC = ([circumference of the affected side—circumference of the unaffected side]/circumference of the unaffected side) × 100%. The highest PEC value among the PEC values at different arm levels was used as the representative PEC^2,23–25^.

### Lymphoscintigraphy acquisition

The lymphoscintigraphy images were obtained at 1 and 2 h after subcutaneous injection of ^99m^Tc-phytate (100 nm, filtered) into the second and/or third web space of both hands with a dose of 37 MBq/0.1 mL per web space. After ^99m^Tc-phytate injection, patients were instructed to exercise (clench and unclench) with rubber balls for 30 min to increase lymphatic flow. Anterior and posterior whole body images were acquired in a 256 × 1024 matrix with the patient in a supine position. One of three different dual-head gamma camera system devices was used: BrightView (Philips Healthcare, Best, Netherlands), Infinia (GE Healthcare, Waukesha, WI, USA), or Symbia Evo Excel (Siemens Healthineers, Erlangen, Germany), with a low-energy high-resolution parallel whole collimator at a scan speed of 13 cm/minute.

### Image interpretation

Whole body lymphoscintigraphy images were independently evaluated in a blind manner by two experienced nuclear medicine physicians. In cases of discrepancy, a consensus evaluation was achieved after discussion. Each image was categorized into stages from 0 to 6 according to the Taiwan lymphoscintigraphy stage proposed by Cheng et al.^16^. Proximal lymph nodes were defined as axillary, infraclavicular, and supraclavicular lymph nodes, and intermediate lymph nodes were defined as collateral nodes found in the upper arm or forearm areas.

### Statistical analysis

Interval and continuous variables are expressed as median with interquartile range (IQR), and dichotomous variables as number with percentage. The change in PEC and lymphoscintigraphic stage was defined as baseline value—follow-up value. The PEC and stage at baseline and follow-up lymphoscintigraphy were compared using the Wilcoxon signed-rank test. Associations between PEC, lymphoscintigraphy stage, and clinical variables were evaluated with Spearman’s rank correlation. Univariable and multivariable linear regression analyses were performed. Independent variables showing *P* values ≤ 0.20 in the univariable analysis were included in the stepwise multivariable analysis. Model selection was based on Akaike’s information criterion. Linear regression assumptions were checked by testing linearity, normality, homoscedasticity, and multicollinearity. The “networkD3” and “car” packages in R software (version 4.1.3 R foundation for Statistical Computing, Vienna, Austria) were used to conduct the statistical analyses.

### Ethics approval

Institutional review board of Seoul Asan medical center approved this study (IRB No. 2022-0869) and waived the need to obtain informed consent because of its retrospective nature. This study was conducted in accordance with the Declaration of Helsinki and our institutional guidelines.

## Results

### Patient characteristics

**Figure 1:**
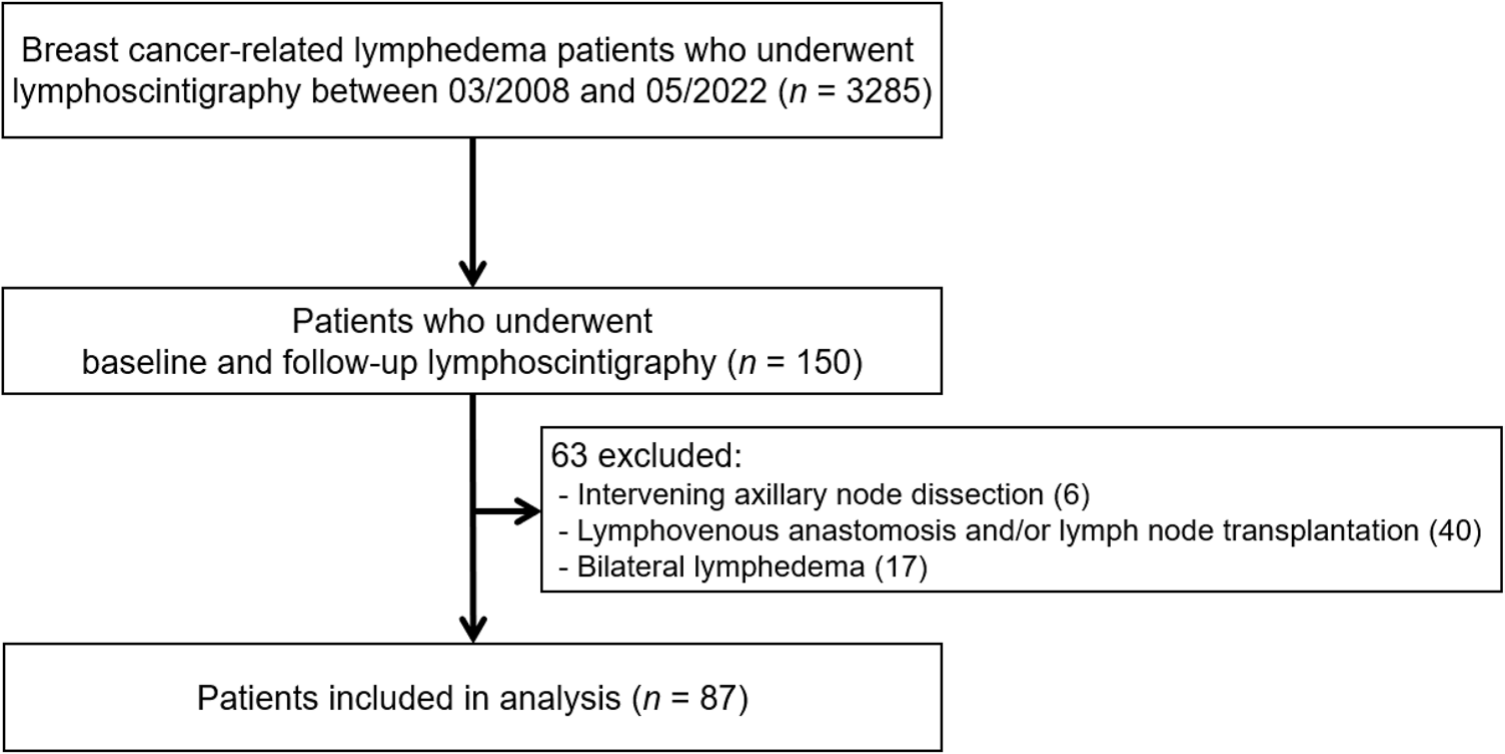
Flow chart of the patient selection process.

Among the initially identified 3285 patients with BCRL who underwent lymphoscintigraphy, 150 patients underwent both baseline and follow-up lymphoscintigraphy during the study period. However, 63 patients were excluded from the analysis for the following reasons: intervening axillary node dissection (*n* = 6), intervening lymphovenous anastomosis and/or lymph node transplantation (*n* = 40), and bilateral lymphedema (*n* = 17). Finally, 87 patients were included in our analysis (Fig. 1). The baseline characteristics of the patients are summarized in Table 1. Information on the T and N stages of breast cancer and the number of dissected lymph nodes was not available for five patients who underwent breast cancer surgery at an outside hospital. All patients included underwent CDT for BCRL. Nodal radiation therapy was performed in 60 (69%) patients, non-nodal radiation therapy in 13 (15%), and radiation therapy with an unknown target field in five patients (6%). Baseline and follow-up lymphoscintigraphy were performed at a median of 7 (IQR: 2‒14) and 78 (IQR: 49‒116) months after surgery, respectively.

**Table 1:** Patient characteristics.

Variables	Median (IQR) or *n* (%)
Age at baseline	48 (43‒55)
Female sex	87 (100%)
BMI at baseline (kg/m^2^)	23.2 (21.7‒26.2)
BMI at follow-up (kg/m^2^)	23.5 (21.3‒26.2)
T stage (Tis/1/2/3/4)*	2/18/44/14/4 (2/21/51/16/5%)
N stage (N0/1/2/3)*	13/32/26/11 (15/37/30/13%)
Surgery	
Breast-conserving operation	40 (46%)
Mastectomy	47 (66%)
Axillary lymph node dissection	75 (86%)
Number of dissected lymph nodes^†^	16 (11‒19)
Nodal radiation	60 (69%)
Chemotherapy	82 (94%)
Cellulitis	23 (26%)
Poor compliance	19 (22%)
Underwent CDT	87 (100%)

*BMI* body mass index, *IQR* interquartile range, *CDT* complex decongestive therapy.

*^, †^Data were not available for five and two patients, respectively.

### Relationship of lymphoscintigraphy stage and PEC

**Figure 2:**
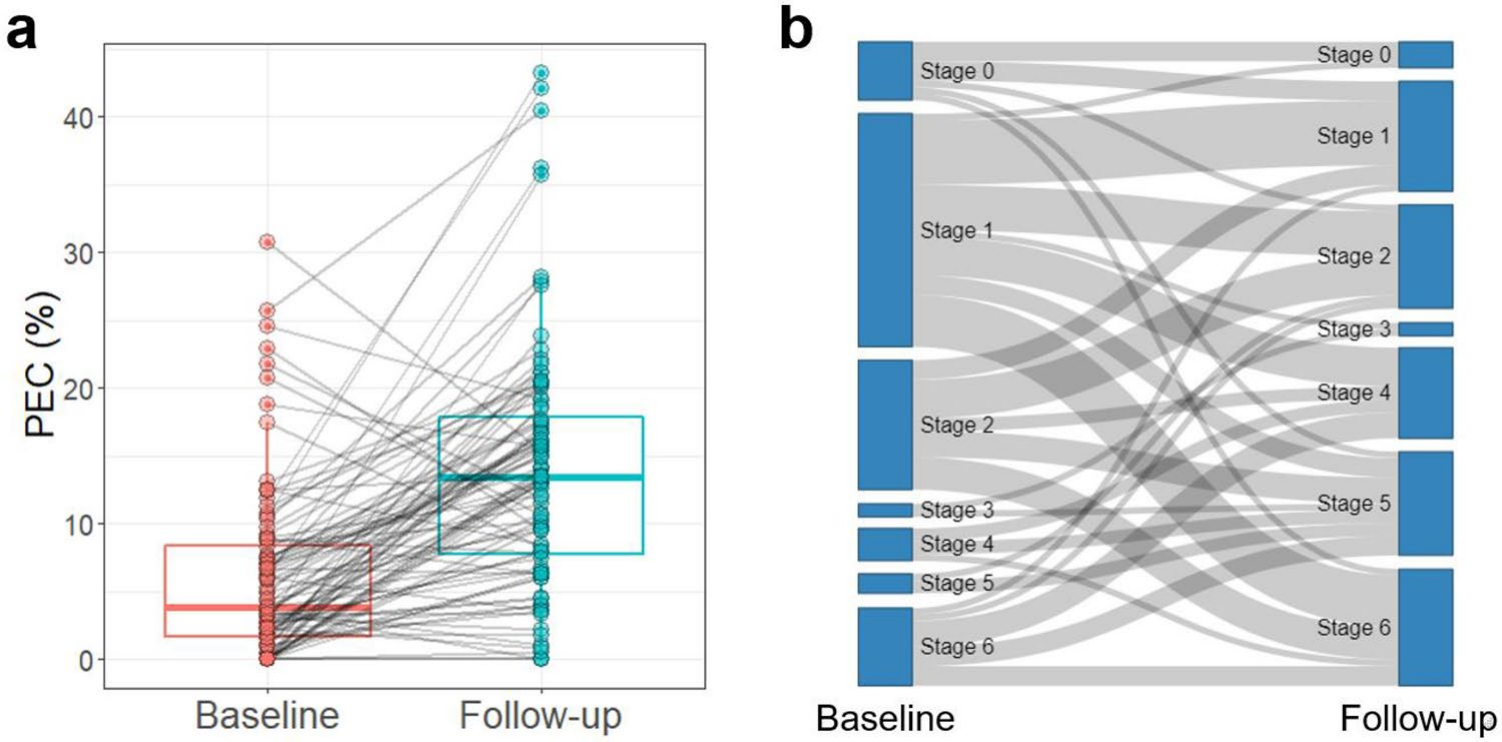
Boxplot showing changes in PEC (**a**) and a Sankey plot illustrating changes in lymphoscintigraphic stage (**b**) between baseline and follow-up. The figure was created using R software (version 4.1.3, R foundation for Statistical Computing, Vienna, Austria).

Both PEC and lymphoscintigraphy stages changed in various manners (Fig. 2). Overall, the median PEC showed a significant increase from 3.8% (IQR: 1.6‒8.3%) at baseline to 13.4% (IQR: 7.7‒17.8%) at follow-up (*P* < 0.001). Lymphoscintigraphy stage also showed a significant increase at follow-up lymphoscintigraphy in comparison with baseline (median [IQR]: 1 [1–3] vs 4 [2–5], *P* < 0.001). Specifically, 34 patients exhibited the disappearance of lymph nodes that were initially present in the baseline image, while three patients showed the emergence of lymph nodes that was not visible in the baseline image during the follow-up. In relation to dermal backflow, nine individuals experienced the complete disappearance of dermal backflow in the follow-up compared to the baseline. In 28 cases, dermal backflow was absent in the baseline but emerged in the follow-up image. Ten patients showed changes in the extent of preexisting dermal backflow from the baseline to the follow-up image.

**Figure 3:**
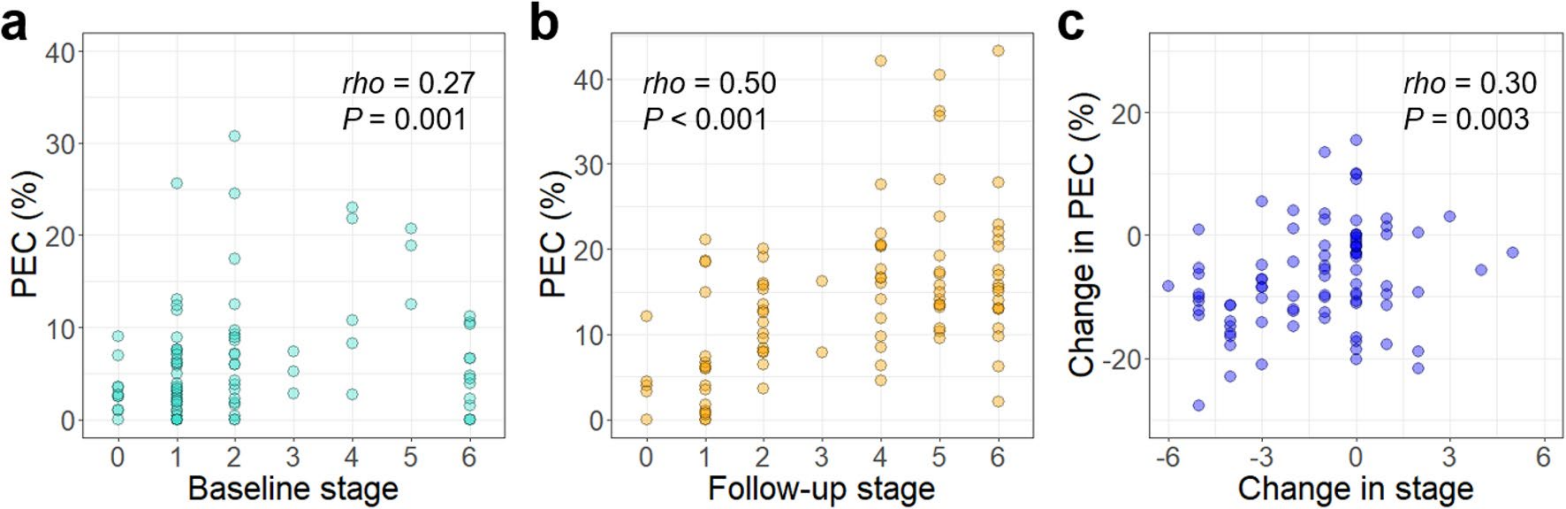
Scatter plots comparing PEC and lymphoscintigraphic stage at baseline (**a**), follow-up (**b**), and change in PEC and change in stage between baseline and follow-up (**c**).

**Figure 4:**
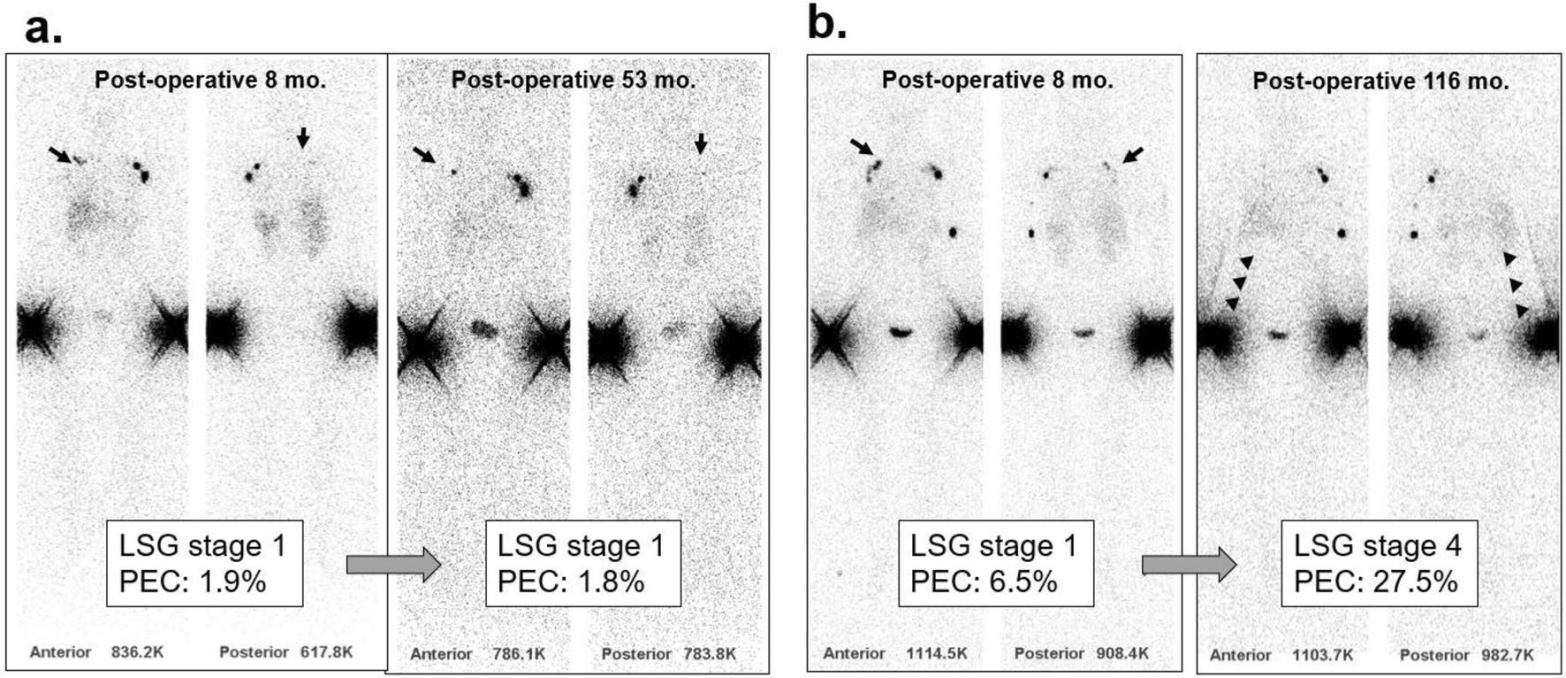
Representative lymphoscintigraphy images of patients who underwent right modified radical mastectomy with axillary lymph node dissection. Baseline lymphoscintigraphy of a 47-year-old woman shows asymmetrically decreased proximal nodal uptake (**a**, black arrow) without dermal backflow. Follow-up lymphoscintigraphy shows a similar pattern to baseline lymphoscintigraphy with stage I at baseline and follow-up, and PECs relatively consistent at 1.9% and 1.8%, respectively. Baseline lymphoscintigraphy of a 58-year-old woman shows asymmetrically decreased axillary nodal uptake (**b**, black arrow) without dermal backflow (stage 1). Follow-up lymphoscintigraphy shows no axillary nodal uptake with new dermal backflow in the forearm (white arrowhead). The PEC showed a substantial increase from baseline (6.5%) to follow-up (27.5%).

The PEC and lymphoscintigraphy stage were positively correlated at both baseline (*rho* = 0.27, *P* = 0.001) and follow-up (*rho* = 0.50, *P* < 0.001) (Fig. 3a and b). The change in PEC and change in lymphoscintigraphy stage between baseline and follow-up also showed a positive correlation (*rho* = 0.30, *P* = 0.003; Fig. 3c). Representative images at baseline and follow-up lymphoscintigraphy are shown in Fig. 4.

### Associations between lymphoscintigraphy stage and clinical factors

The lymphoscintigraphy stages at both baseline and follow-up scans were positively correlated with the time interval from surgery (baseline: *rho* = 0.26, *P* = 0.016; follow-up: *rho* = 0.34, *P* = 0.001). However, other clinical factors were not associated with baseline lymphoscintigraphy stage (Table 2).

**Table 2:** Comparison of lymphoscintigraphy stages at baseline and follow-up according to patient characteristics.

Variables	Baseline stage		Follow-up stage	
	Median (IQR)	*P*	Median (IQR)	*P*
BMI (kg/m^2^)		0.796		0.101
> median	1 (1‒2)		2 (1‒5)	
≤ median	1 (1‒4)		4 (2‒5)	
Number of dissected lymph nodes		0.451		0.143
> median	1 (1‒3)		4 (2‒6)	
≤ median	1 (1‒2)		3 (1‒5)	
Type of axillary dissection		0.116		0.023
ALND	1 (1‒3)		4 (2‒5)	
SNB	1 (0‒2)		1 (0‒4)	
Radiation therapy		0.107		0.003
Nodal	2 (1‒4)		4 (2‒5)	
None or non-nodal	1 (1‒2)		2 (1‒4)	
Chemotherapy (vs none)		0.201		0.133
Done	1 (1‒3)		4 (2‒5)	
Not done	2 (2‒4)		2 (1‒4)	
Bandage compression		0.623		< 0.001
Required	2 (1‒4)		5 (2‒6)	
Not required	1 (1‒2)		1 (1‒4)	
Cellulitis		0.590		0.024
Yes	2 (1‒2)		4 (4‒5)	
None	1 (1‒4)		2 (1‒5)	
Compliance		0.764		0.368
Poor	1 (1‒4)		4 (2‒5)	
Good	1 (1‒2)		4 (1‒5)	

*ALND* axillary lymph node dissection, *IQR* interquartile range, *NA* not applicable, *SNB* sentinel node biopsy.

The lymphoscintigraphy stage at follow-up was significantly different according to type of axillary node dissection (*P* = 0.023), type of radiation therapy (*P* = 0.003), bandage compression (*P* < 0.001), and cellulitis (*P* = 0.024) (Table 2). The change in stage between baseline and follow-up lymphoscintigraphy was significantly associated with chemotherapy (*P* = 0.046), bandage compression (*P* < 0.001), and cellulitis (*P* = 0.009; Supplementary Table 1).

### Linear regression analyses of factors associated with PEC

In the univariable regression analyses, the interval between surgery and lymphoscintigraphy, nodal radiation, bandage compression, cellulitis, poor compliance, lymphoscintigraphy stage at baseline, lymphoscintigraphy stage at follow-up, and change in lymphoscintigraphy stage were significantly associated with the PEC at follow-up (Table 3). The stepwise multivariable analysis revealed that lymphoscintigraphy stage at follow-up (adjusted *β* = 1.47 [95% CI: 0.67‒2.28], *P* = 0.001) in addition to cellulitis (adjusted *β* = 4.34 [95% CI: 0.77‒7.91], *P* = 0.018) were independent variables associated with PEC at follow-up, however, baseline lymphoscintigraphy stage was not (adjusted *β* = 0.63 [95% CI: − 0.17‒1.43],* P* = 0.121). Poor compliance was significant factor in univariable analysis for follow-up lymphoscintigraphy; however, it was not a significant factor in the multivariable analysis. The results of the univariable and multivariable linear regression analyses for the changes in PEC are shown in Supplementary Table 2.

**Table 3:** Results of univariable and multivariable linear regression analyses for PEC at follow-up lymphoscintigraphy.

Variables	Univariable		Multivariable	
	Crude *β* (95% CI)	*P*	Adjusted *β* (95% CI)	*P*
Interval between surgery and lymphoscintigraphy (mo.)	0.04 (0.01‒0.07)	0.024	Not included	
BMI (kg/m^2^)	0.11 (− 0.41‒0.64)	0.673		
ALND (vs SNB)	4.88 (− 0.44‒10.19)	0.072	Not included	
Nodal radiation (vs none/non-nodal)	5.19 (1.40‒8.97)	0.008	Not included	
Chemotherapy (vs none)	7.11 (− 1.35‒15.57)	0.098	Not included	
Bandage compression at follow-up (vs not)	4.28 (0.70‒7.86)	0.020	Not included	
Cellulitis (vs none)	4.97 (1.04‒8.89)	0.014	4.34 (0.77‒7.91)	0.018
Poor compliance (vs not)	5.68 (1.50‒9.86)	0.008	2.96 (− 0.57‒6.49)	0.099
Lymphoscintigraphy stage at baseline	0.98 (0.05‒1.90)	0.039	0.63 (− 0.17‒1.43)	0.121
Lymphoscintigraphy stage at follow-up	1.98 (1.19‒2.78)	< 0.001	1.47 (0.67‒2.28)	0.001
Change in lymphoscintigraphy stage	− 0.84 (− 1.61‒ − 0.07)	0.033	Not included	

*ALND* axillary lymph node dissection, *BMI* body mass index, *CI* confidence interval, *SNB* sentinel node biopsy.

## Discussion

In this study, we assessed changes on long-term follow-up lymphoscintigraphy and their relationship with clinical features in patients with BCRL. The clinical course of lymphedema and the lymphoscintigraphy findings showed a variety of changes over time, with a general deterioration. The lymphoscintigraphy stage was closely associated with the clinical severity of lymphedema assessed by PEC at baseline and follow-up, as well as their changes. In the multivariable analysis, lymphoscintigraphy stage at follow-up was found to be an independent variable for PEC, whereas lymphoscintigraphy stage at baseline was no longer significantly associated with PET at follow-up. In addition to initial lymphoscintigraphy for diagnostic purposes, our results suggest that lymphoscintigraphic remapping may be useful for monitoring functional changes in BCRL during follow-up.

Our results regarding changes in lymphoscintigraphic findings are consistent with a previous report by Szuba et al.^22^ who examined serial lymphoscintigraphies at 1‒6 weeks, 1 year, and 2 years after axillary lymph node dissection in patients with breast cancer. They descriptively reported that loss of previously functional lymph nodes and appearance of new dermal backflow were commonly observed in patients with BCRL, which can be translated into change to a higher lymphoscintigraphic stage during follow-up. In addition, our study revealed that such changes in functional lymphatics visualized on lymphoscintigraphy are closely related to the changes in clinical manifestation of BCRL during follow-up. Our longer follow-up period of a median of 78 months after surgery is also a characteristic of our study differentiating it from that of Szuba et al.^22^. Our findings suggest that long-term changes in the lymphatic system occur over time and can be accurately tracked by follow-up lymphoscintigraphy. We also found that lymphoscintigraphy stage at follow-up showed higher degree of correlation with clinical severity in terms of PEC and significant associations with known clinical risk factors such as axillary nodal dissection, nodal radiation therapy^22^ and cellulitis^26,27^, which is consistent with previous reports. However, we did not find such relationships on baseline lymphoscintigraphy. Early lymphedema can occur without lymphoscintigraphic evidence of lymphatic dysfunction^22^. Possible mechanisms of pathophysiology in BCRL include absent or insufficient collateral lymphatic circulation, lymphatic pump failure induced by lymphatic overload, and venous hypertension^28^. It is plausible that early baseline lymphoscintigraphy could not sufficiently reflect such pathophysiology, which may develop over the course of the disease. For patients with persistent BCRL whose imaging finding does not align well with clinical presentation, lymphoscintigraphy remapping may be required for comprehensive assessment and optimal management decision.

In our study, we performed subcutaneous injection of ^99m^Tc-phytate for lymphoscintigraphy acquisition. Yet to date, preference between intradermal and subcutaneous injection for lymphoscintigraphy acquisition has not been strictly standardized, even in the recent 2020 consensus document of the International Society of Lymphology^29^. In some studies, suggested that intradermal injection is associated with rapid lymphatic transport^30^, and has favorable tracer kinetics for evaluating the superficial lymphatic system draining into the skin and subcutaneous tissue^31–33^. However, subcutaneous injection is known for its high reliability in diagnosing lymphedema^34^. Our study analyzed lymphoscintigraphy based on lymphoscintigraphy stage system proposed by Cheng et al., which also employed subcutaneous injection of ^99m^Tc-phytate^16^. Additionally, most studies evaluating the severity of lymphedema through lymphoscintigraphy have used subcutaneous injection^17,35,36^. Therefore, lymphoscintigraphy conducted with subcutaneous injection of ^99m^Tc-phytate could be beneficial for assessing severity and monitoring of lymphedema patients.

Several staging systems for lymphoscintigraphy in patients with upper arm lymphedema have been proposed, including a previous study with ^99m^Tc-phytate by Cheng et al. (Taiwan lymphoscintigraphy stage)^16^, and a study with ^99m^Tc-labeled human serum albumin by Mikami et al.^17^. Although both staging systems are similar, they do have some distinct features. Mikami’s classification consists of five stages, mainly differentiated by the presence and extent of dermal backflow. In the Taiwan lymphoscintigraphy staging system, lymphoscintigraphy stages are largely divided into partial obstruction (stages 1–3) and total obstruction (stage 4–6) according to the presence of proximal or intermediate lymph node uptake, with further subdivision based on the presence or extent of dermal backflow. The presence of a functional proximal lymph node on lymphoscintigraphy is known to be associated with a lower frequency of lymphedema development^37^, less severe clinical manifestation, and favorable response to complex decongestive therapy^15^. We speculate that the Cheng staging system better depicted the variable clinical aspects of lymphedema in our study population who underwent lymphoscintigraphy with ^99m^Tc-phytate. Both staging systems lack a clear anatomical definition of proximal lymph nodes. Of note, the hierarchy of dermal backflow differs between the two staging systems: the presence of dermal backflow limited to the forearm is regarded as a higher stage than that involving the upper arm or entire arm in Mikami’s classification, whereas the presence of dermal backflow limited to the forearm is considered to represent less severe disease status than that in the entire arm according to the Taiwan lymphoscintigraphy staging system. In our study, we found that the PEC differed substantially between the partial (stage 1‒3) and total obstruction (stage 4‒6) groups, as shown in Fig. 3, whereas differences in PEC within these groups were not observed. The question of which lymphoscintigraphy staging system better depicts BCRL remains unclear, and therefore further studies on this issue are warranted.

There are several limitations to this study. First, there might be selection bias because our study population underwent follow-up lymphoscintigraphy, which might not be performed for every patient with BCRL. Therefore, follow-up lymphoscintigraphy may not be equally effective in all BCRL patients. Further research is needed to elucidate specific patient indication for lymphoscintigraphic remapping. Second, the time interval between baseline and follow-up lymphoscintigraphy, as well as the duration from surgery to baseline lymphoscintigraphy, differed from patient to patient, and therefore an optimal timepoint for follow-up lymphoscintigraphy could not be derived from our study. Third, the type of conservative treatment varied because patients received individually tailored optimal management for lymphedema. While all patients included in the study received CDT, consisting of various combinations of manual lymphatic drainage (MLD), compression, exercise, and skin care, the specific details of CDT received by each patient might differ. In addition, the long-term outcome could be influenced based on the adherence to compression after implementation of CDT. Furthermore in our study, patients who underwent surgical interventions such as lymphovenous anastomosis or lymph node transfer that could directly impact lymphatic flow were excluded. These surgical interventions are progressively gaining ground in the management of lymphedema patients. Research investigating changes before and after treatment and monitoring treatment outcomes through lymphoscintigraphy in patients who have undergone surgery is currently lacking, and further studies are needed in this regard. The pattern of change in lymphoscintigraphy may differ for BCRL patients who have underwent surgical treatment. Fourth, our results may not be applicable to lymphoscintigraphy with different tracers and acquisition protocols, which can substantially affect its interpretation^38^. The procedural standardization of lymphoscintigraphy may enhance the applicability of study results^39,40^. Finally, although our study showed potential clinical value of lymphoscintigraphic remapping, we could not demonstrate that patient care has directly improved from it. Nevertheless, lymphoscintigraphic remapping may enable a tailored rehabilitation program and optimal management based on an understanding of functional changes in lymphatic drainage in patients with BCRL. It is important to interpret these findings cautiously, recognizing the need for further research to establish a direct link between lymphoscintigraphic remapping and improvement of patient outcomes. In conclusion, the clinical course of persistent BCRL, its pattern on lymphoscintigraphy, and its clinical features varied over time. Lymphoscintigraphy stages at follow-up were found to be associated with clinical risk factors including axillary nodal dissection, nodal radiation therapy, cellulitis and were identified as independent variables for the current clinical severity assessed by PEC, while baseline lymphoscintigraphy stages were not. In addition to initial lymphoscintigraphy for diagnostic purposes, lymphoscintigraphic remapping during follow-up can objectify and visualize diverse changes in the functioning of the lymphatic system, which may help categorize the clinical severity of lymphedema and guide the optimal management plan in patients with BCRL.

### Supplementary Information

Supplementary Tables.

## Data Availability

The datasets used and/or analyzed during the current study are available from the corresponding author on reasonable request.

## Abbreviations

BCRL: Breast cancer-related lymphedema
BIA: Bioelectrical impedance analysis
CI: Confidence interval
CT: Computed tomography
IQR: Interquartile range
IRB: Institutional review board
MRI: Magnetic resonance imaging
PEC: Percentage of excessive circumference
