# Supplementary material for: Lymphatic remapping by long-term lymphoscintigraphy follow-up in secondary lymphedema after breast cancer surgery
Source: Sci Rep. 2024 Jan 6;14:728. doi: 10.1038/s41598-023-50558-7 (PMC10771519; doi:10.1038/s41598-023-50558-7)
Supplement: Supplementary file 1 — Supplementary Tables. [file 41598_2023_50558_MOESM1_ESM.docx]

**Supplementary Online Contents**

**Lymphatic remapping by long-term lymphoscintigraphy follow-up in secondary lymphedema after breast cancer surgery (by Garam Hong et al.)**

**Supplementary Table 1.** Comparison of the change in lymphoscintigraphic stage between baseline and follow-up according to patient characteristics.

**Supplementary Table 2.** Results of univariable and multivariable linear regression analyses for changes in PEC.

**Supplementary Table 1.** Comparison of the change in lymphoscintigraphic stage between baseline and follow-up according to patient characteristics.

| Variables | Median (IQR) | *P* |
| --- | --- | --- |
| Change in BMI (kg/m^2^) |  | 0.389 |
| > median | −1 (−3‒0) |  |
| ≤ median | 0 (−3‒0) |  |
| Number of dissected lymph nodes |  | 0.351 |
| > median | −1 (−3‒0) |  |
| ≤ median | 0 (−2‒0) |  |
| Type of axillary dissection |  | 0.169 |
| ALND | −1 (−3‒0) |  |
| SNB | 0 (−1‒1) |  |
| Radiation therapy |  | 0.165 |
| Nodal | −1 (−4‒0) |  |
| None or non-nodal | 0 (−1‒0) |  |
| Chemotherapy (vs none) |  | 0.046 |
| Done | −1 (−3‒0) |  |
| Not done | 1 (0‒1) |  |
| Bandage compression |  | <0.001 |
| Required | −2 (−4‒0) |  |
| Not required | 0 (−1‒1) |  |
| Cellulitis |  | 0.009 |
| Yes | −3 (−3‒−1) |  |
| None | 0 (−3‒0) |  |
| Compliance |  | 0.676 |
| Poor | −1 (−3‒0) |  |
| Good | −1 (−3‒0) |  |

ALND = axillary lymph node dissection, IQR = interquartile range, NA = not applicable, SNB = sentinel node biopsy.

**Supplementary Table 2.** Results of univariable and multivariable linear regression analyses for changes in PEC.

|  | Univariable |  |  | Multivariable |  |
| --- | --- | --- | --- | --- | --- |
| Variables | Crude *β* (95% CI) | *P* |  | Adjusted *β* (95% CI) | *P* |
| Interval between baseline and follow-up lymphoscintigraphy (mo.) | −0.05 (−0.10‒0.00) | 0.049 |  | 0.00 (0.00‒0.00) | 0.170 |
| Change in BMI (kg/m^2^) | −0.52 (−1.60‒0.55) | 0.335 |  |  |  |
| ALND (vs SNB) | −5.86 (−11.52‒−0.20) | 0.043 |  | Not included |  |
| Nodal (vs no/non-nodal) radiation | −6.60 (−10.7‒−2.50) | 0.002 |  | −4.05 (−8.41‒0.30) | 0.068 |
| Chemotherapy (vs none) | −13.95 (−22.74‒−5.16) | 0.002 |  | −8.57 (−18.68‒1.54) | 0.095 |
| Band compression at follow-up (vs none) | −1.06 (−5.08‒2.96) | 0.600 |  |  |  |
| Cellulitis (vs none) | −0.44 (−4.87‒3.99) | 0.845 |  |  |  |
| Poor compliance (vs none) | −3.26 (−7.97‒1.45) | 0.172 |  | Not included |  |
| Change in lymphoscintigraphy stage | 1.12 (0.29‒1.94) | 0.009 |  | 0.77 (−0.03‒1.57) | 0.059 |

ALND = axillary lymph node dissection, BMI = body mass index, CI = confidence interval, SNB = sentinel node biopsy
